# Physical health and ecological momentary assessments during COVID-19: Data from the ‘Corona Health’ app users

**DOI:** 10.1016/j.dib.2025.111289

**Published:** 2025-01-13

**Authors:** Johannes Allgaier, Felizitas Eichner, Stefan Störk, Peter Heuschmann, Rüdiger Pryss

**Affiliations:** a Institute of Medical Data Science, University Hospital Würzburg, 97070, Germany; b Institute of Clinical Epidemiology and Biometry, University of Würzburg, 97080, Germany; c Dept. Clinical Research & Epidemiology, Comprehensive Heart Failure Center Würzburg, University Hospital Würzburg, 97078 Würzburg, Germany; d Clinical Trial Center, University Hospital Würzburg, 97070, Germany

**Keywords:** Public health monitoring, Longitudinal studies, Behavioral data, Digital health interventions

## Abstract

The dataset published in this work is derived from the ‘Corona Health’ app, developed in collaboration with the German Robert Koch Institute (RKI) during the early stages of the COVID-19 pandemic. The smartphone application aimed to monitor the mental and physical health of the public through real-time data collection. The dataset incorporates Ecological Momentary Assessments (EMA), Patient-reported Outcome Measures (PROMs), GPS data, and digital phenotyping from app users who consented. The data includes responses from 1805 mostly German users who completed baseline and follow-up questionnaires, capturing their physical health status over time. These questionnaires cover health-related topics, including medical history, cardiovascular risk factors, lifestyle habits, and the impact of the pandemic on health behaviors. The resulting dataset offers insights into health trajectories and behaviors during the pandemic and can be utilized for further research on physical health, user engagement, and the efficacy of EMA and digital phenotyping in health monitoring. The data is publicly available under a Creative Commons license on zenodo.org/records/11093394.

Specifications Table

**Table:** 

Subject	Public Health, Digital Health Monitoring
Specific subject area	Health data collection during COVID-19 using smartphone-based applications.
Type of data	Questionnaire responses (baseline and follow-up); geospatial data; device usage statistics; digital phenotyping data (processed and raw).
Data collection	Data were collected through the Corona Health app using Ecological Momentary Assessments (EMA) and Patient-Reported Outcome Measures (PROMs). The app recorded user input via structured questionnaires and passive sensor data, including GPS and app usage. Questionnaires were derived from validated instruments and translated into eight languages.
Data source location	Predominantly Germany (98.7% of participants); data stored in Zenodo repository under DOI: 10.5281/zenodo.11093394.
Data accessibility	Repository: Zenodo; DOI: 10.5281/zenodo.11093394. Data available under Creative Commons license.
Related research article	Beierle, F., et al. (2021). Corona health—a study-and sensor-based mobile app platform exploring aspects of the COVID-19 pandemic. *Int. J. Environ. Res. Public Health*, 18, 7395.

Specifications table summarizing the key aspects of the data published in the ‘Corona Health’ study.

## 1 Value of the Data

• The data collected through the Corona Health app offer significant insights into the physical and mental health impacts of the COVID-19 pandemic, particularly among users from Germany [1]. Leveraging Ecological Momentary Assessment (EMA) and Patient-Reported Outcome Measures (PROMs), the app captures real-time, longitudinal data that reduce recall bias and provide a dynamic understanding of health behaviors and well-being during the pandemic from 2020-07 until 2023-10.
• This convenience sample of predominantly German users allows for in-depth analysis of regional variations in health outcomes and engagement with health interventions. The dataset includes a wide range of information, from baseline demographics and physical health metrics to mobile sensing data such as GPS locations. This comprehensive approach enables the exploration of behavioral and environmental factors influencing health during a global crisis.
• Despite the inherent limitations of a convenience sample, the breadth and depth of the data provide valuable opportunities for understanding the pandemic's localized impacts and for informing public health strategies. The rigorous data collection methods, adherence to ethical standards, and inclusion of validated questionnaires ensure the dataset's reliability and applicability for future research. This makes the Corona Health dataset a crucial resource for exploring the long-term effects of the pandemic and advancing digital health research.

## 2 Background

In the face of the unprecedented challenges posed by the early stages of the COVID-19 pandemic in 2020, our research team, in collaboration with the German Robert Koch Institute (RKI), developed an innovative smartphone application named ‘Corona Health´1. This application was designed to enable the public to actively participate in a series of studies aimed at monitoring mental and physical health during the pandemic. Through this initiative, we sought to capture real-time data (aka ecological data) on how various pandemic-related events—such as government-imposed lock-downs and social restrictions—affected individual well-being. Corona Health mainly integrates EMA techniques with PROMs [2], Mobile Crowdsensing [2] and Digital Phenotyping [3], which are crucial for gathering accurate in-the-moment data and overcoming the limitations typically associated with retrospective survey methods. Such traditional methods are often limited by recall bias, making them less reliable for understanding the dynamic changes in mental states and lifestyle over time. Our application allows users to complete baseline and follow-up questionnaires that gauge their mental and physical health status, facilitating a longitudinal study design that provides valuable insights into health trajectories during the pandemic. In addition, Corona Health offers GPS measurements and Digital Phenotyping measurements if users allowed them to be recorded.

**Fig. 1:**
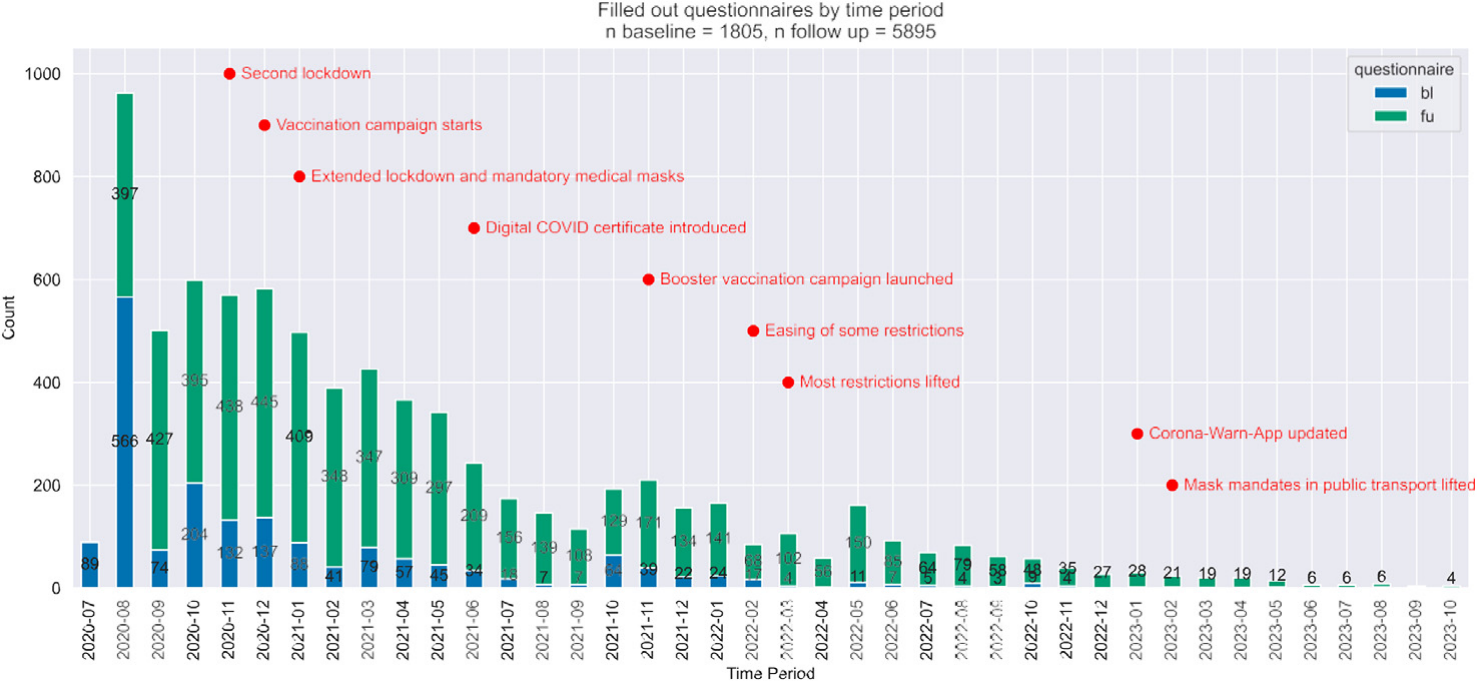
Created questionnaires over time. Red text indicates the chronology of the pandemic in Germany [4]. The highest level of interest was at the start of the pandemic, and no new users have joined since 2023.

In this work, we publish the data collected on the study ‘physical health of adults’ from the Corona Health application. This dataset contains 1805 users contributing 1805 baseline questionnaires and 5895 follow-up questionnaires between 2020-07 and 2023-10 (see Fig. 1). An overview on data collection is shown in Table 1.

**Table 1:** Demographics and some key numbers about the baseline questionnaire. Std = Standard deviation.

Key Facts	
No. of questionnaires	1805 Baseline + 5895 Follow-up
n Tracking Consent GPS (ratio)	1366 (75%)
n Tracking Consent App Usage (ratio)	101 (5.6%)
Sociodemographics	
Age, mean (SD)	41.7 (15.1)
Gender Ratio	36% Male | 64% Female | 0% Diverse
Body Mass Index, mean (SD)	26.7 (6.23)
Users located in Germany (ratio)	98.7%
Lifestyle habits at baseline	
Daily Smokers (ratio)	292 (16.2%)
Daily fruit consumers before lockdown (ratio)	533 (29.5%)
Daily fruit consumers after lockdown (ratio)	538 (29.8%)
Daily vegetable consumers before lockdown (ratio)	562 (31.1%)
Daily vegetable consumers after lockdown (ratio)	559 (31.0%)
Cardiovascular Health at baseline	
History of hypertension (ratio)	454 (25.2%)
History of diabetes mellitus (ratio)	113 (6.3%)
History of hyperlipidemia (ratio)	461 (25.5%)

## 3 Experimental Design, Materials and Methods

This section outlines the app's development, study structure, data collection methods, ethical considerations, and technical architecture.

### 3.1 Platform development and deployment

The *Corona Health* app, built on the PHP-based *TrackYourHealth* framework [5], was released in July 2020 on Android and iOS platforms and supported in eight languages. Development incorporated interdisciplinary collaboration, regulatory compliance, and established frameworks, ensuring robust data quality and ethical standards.

Experts across multiple disciplines, including physicians and data scientists, collaborated to ensure the app's methodological rigor. The app adhered to EU Medical Device Regulation standards, achieving high levels of data protection and user safety. Ecological Momentary Assessment (EMA) methods were integrated to reduce recall bias, improving data accuracy, while sensor and digital phenotyping techniques enabled the collection of objective data, such as location, to complement subjective self-reports.

### 3.2 Data collection methodology

Data were collected via user input and automated sensing upon user consent: An active input from users who completed questionnaires, and passive sensing, where we automatically collected data including device type, OS, location (11.1 km accuracy), and app usage on Android devices.

### 3.3 Technical implementation and data handling

The app utilized a relational database within the *TrackYourHealth* backend to manage structured, multilingual data.

**Questionnaire pipeline** The development of questionnaires began with designing questions in Microsoft Excel, where metadata, question types, and language options were specified. These Excel files were then converted to JSON format using Python and stored in the backend, making them accessible via a RESTful API. This setup allowed for dynamic deployment, enabling real-time updates within the app. Data exchange followed the JSON protocol, ensuring structured and efficient data handling for both deploying and submitting questionnaire data.

The app was made available in eight languages on major app stores, and upon initial launch, users encountered a mandatory disclaimer along with onboarding prompts that covered notification and data permissions. Corona Health's architecture supported both online and offline functionality, ensuring uninterrupted data collection. User data were managed within a relational database using SQL to maintain organization and data integrity.

## 4 Data Description

The datasets and codebook are available under the Creative Commons license on zenodo.org/records/11093394. Table 2 provides a detailed description of all questions from the two questionnaires and is also available on Zenodo (codebook.xlsx). Additionally, Zenodo hosts the raw data, which includes both baseline and follow-up questionnaires, as well as the flattened and separated datasets for baseline and follow-up.

**Table 2:** Codebook of the two questionnaires baseline and follow-up. Because these questionnaires share a major part of their questions, we provide these here within one table. A enriched codebook is provided on GitHub/joa24jm/CH-Heart.

Questiontype	Required	Label	In Follow- Up?	Question	Encoding and Answeroptions
Knob	true	Alter	No	How old are you (in years) ?	Integer
SingleChoice	true	Geschlecht	No	Which gender are you?	0=Female, 1=Male, 2=Transgender
SingleChoice Knob	true	Country	No	In which country do you currently live?	Knob to chose countrynames from
SingleChoice	true	heart1	No	Coronary heart disease or Angina Pec toris (Similar in CDC Logo Behav- ioral Risk Factor Surveillance System (BRFSS) questionnaire [6])	0=No, 1=Yes, 2=I don't know
SingleChoice	true	heart2	No	Heart failure	0=No, 1=Yes, 2=I don't know
SingleChoice	true	heart3	No	Myocardial infarction (Similar in BRFSS [6])	0=No, 1=Yes, 2=I don't know
SingleChoice	true	Fibrl	No	Atrial fibrillation	0=No, 1=Yes, 2=I don't know
SingleChoice	true	Stroke	No	Stroke or its preliminary stage (a so called “TIA”) (Similar in BRFSS [6])	0=No, 1=Yes, 2=I don't know
SingleChoice	true	Charc	No	Peripheral vascular disease and periph eral arterial occlusive disease	0=No, 1=Yes, 2=I don't know
SingleChoice	true	Asthm	No	Asthma, including allergic asthma (Similar in BRFSS [6])	0=No, 1=Yes, 2=I don't know
Questiontype		Label	In Follow- Up?	Question	Encoding and Answeroptions
SingleChoice	true	Bronc	No	Chronic bronchitis, chronic obstructive lung disease or lung emphysema (Simi- lar in BRFSS [6])	0=No, 1=Yes, 2=I don't know
SingleChoice	true	Pneum	No	Pneumonia (Similar in National Health Interview Survey (NHIS) [7])	0=No, 1=Yes, 2=I don't know
SingleChoice	true	Lungd	No	Other lung diseases	0=No, 1=Yes, 2=I don't know
SingleChoice	true	Rheum	No	Rheumatism/rheumatic disease	0=No, 1=Yes, 2=I don't know
SingleChoice	true	liver1	No	Mild liver dysfunction	0=No, 1=Yes, 2=I don't know
SingleChoice	true	liver2	No	Severe liver dysfunction	0=No, 1=Yes, 2=I don't know
SingleChoice	true	Kidne	No	Chronic kidney disease or kidney failure (Similar in NHIS [7])	0=No, 1=Yes, 2=I don't know
SingleChoice	true	Ulcer	No	Gastrointestinal ulcer disease	0=No, 1=Yes, 2=I don't know
SingleChoice	true	Tumou	No	Cancer/Tumor (Similar in NHIS [7])	0=No, 1=Yes, 2=I don't know
SingleChoice	true	Hearing	No	Difficulties with hearing, such as listen- ing to speech in a noisy environment	0=No, 1=Yes, 2=I don't know
SingleChoice	true	Hyperacu	No	Complaints caused by external noise sources, which were too loud or felt un- comfortable for you, but which others perceived as normal	0=No, 1=Yes, 2=I don't know
SingleChoice	true	Tinnitus	No	Tinnitus (e.g. rustling, ringing or whistling in the ears without there be- ing an external noise source)	0=No, 1=Yes, 2=I don't know
SingleChoice	true	Vertigo	No	Dizziness	0=No, 1=Yes, 2=I don't know
SingleChoice	true	Smell	No	Disturbance of the sense of smell (Similar in NHIS [7])	0=No, 1=Yes, 2=I don't know
SingleChoice	true	Taste	No	Disturbance of the sense of taste (Similar in NHIS [7])	0=No, 1=Yes, 2=I don't know
SingleChoice	true	smoke1	Yes	Are you currently smoking tobacco products (e.g. cigarettes, cigars, pipe or other tobacco products)? (Simi- lar in Global Adult Tobacco Survey (GATS)10)	1 = Yes, daily, 2 = Yes, from time to time, 3 = No, not anymore, 4 = No, I never smoked, 99 = No response
SingleChoice	true	smoke2	Yes	Has your smoking behavior changed since the lockdown has been imposed mid of march? (Similar in (GATS)[10])	1 = Yes, I smoke more than before, 2 = Yes, I smoke less than before, 3 = No, I smoke about the same amount as before, 4 = I do not smoke, 99 = No respone
SingleChoice	true	alcoh1	No	How often did you drink alcohol in the past 12 months, such as beer, wine, sparkling wine, schnapps, liquor, hard liquor, cocktails, alcoholic mixed drinks or self-made alcoholic products?	1 = Daily or almost daily, 2 = 5-6 days a week, 3 = 3-4 days a week, 4 = 1-2 days a week, 5 = 2-3 daysa month, 6 = Once in a month, 7 = Less than once per month, 8 = Not in the past 12 months, as I don't drink alcohol anymore, 9 = Never or only a few sips in my life
SingleChoice	true	alcoh2	Yes	Has your drinking behavior changed since the lockdown has been imposed mid of march? (Similar in Social Impact of COVID-19 Survey (SICS)?)	1 = Yes, I drink more alcohol, 2 = Yes, I drink less alcohol, 3 = No, I drink about the same amount as before, 4 = I do not drink alcohol, 99 = No respone
SingleChoice	true	alcoh3	Yes	How many drinks containing alcohol do you have on a typical day when you are drinking? [8]	0 = I never drink alcohol, 1 = 1 or 2, 2 = 3 or 4, 3 = 5 or 6, 4 = 7 to 9, 5 = 10 or more, 99 = No response
SingleChoice	true	fruit1	No	How often did you eat fruit before the lockdown has been imposed? Dried, frozen and canned fruit should be in- cluded. Please do not consider fruit juices.	1 = Daily or several times daily, 2 = 4 to 6 times per week, 3 = 1 to 3 times per week, 4 = Less than once per week, 5 = Never
Questiontype	Required	Label	In Follow- Up?	Question	Encoding and Answeroptions
SingleChoice	true	fruit2	Yes	How often do you eat fruit since the lock down has been imposed mid of March?	1 = Daily or several times daily, 2 = 4 to 6 times per week, 3 = 1 to 3 times per week, 4 = Less than once per week, 5 = Never
SingleChoice	true	fruit3	Yes	How has your diet with regard to fruit changed since the lockdown has been imposed mid of March?	1 = I eat more fruit, 2 = I eat less fruit, 3 = I eat the same amount of fruit as before
SingleChoice	true	veget1	No	How often did you eat vegetables or salad before the lockdown has been im- posed? Dried, frozen and canned vegeta- bles should be included. Please do not consider potatoes and vegetable juices.	1 = Daily or several times daily, 2 = 4 to 6 times per week, 3 = 1 to 3 times per week, 4 = Less than once per week, 5 = Never
SingleChoice	true	veget2	Yes	How often do you eat vegetables or salad since the lockdown has been imposed mid of March?	1 = Daily or several times daily, 2 = 4 to 6 times per week, 3 = 1 to 3 times per week, 4 = Less than once per week, 5 = Never
SingleChoice	true	veget3	Yes	How has your diet with regard to fresh or frozen vegetables and salad changed since the lockdown has been imposed mid of March?	1 = I eat more vegetables, 2 = I eat less vegetables, 3 = I eat the same amount of vegetables as before
MultipleChoice	true	eatin1	Yes	How has your diet changed in general since the lockdown has been imposed mid of March?	1 = I eat more often, 2 = I eat bigger portions, 3 = I eat less often, 4 = I eat smaller portions, 5 = I have the feeling my diet is less healthy than before, 6 = I have the feeling my diet is more healthy than before
SingleChoice	true	fastf1	Yes	Have you been eating fast food more frequently since the lockdown?	0 = No, 1 = Yes, 77 = I don't know
SingleChoice	true	fastf2	Yes	Have you been eating frozen food more frequently since the lockdown, for ex- ample frozen pizza, fish sticks or fries?	0 = No, 1 = Yes, 77 = I don't know
SingleChoice	true	sport1	No	How much time did you spend in a typi cal week doing sport, fitness or physical activity in your free time before the lock- down? (in hours)	0 = 1 hour or less, 1 = >1 to 2 hours, 2 = >2 to 3 hours, 3 = >3 to 4 hours, 4 = more than 4 hours, 77 = I don't know
SingleChoice	true	sport2	Yes	How much time do you spend in a typi cal week doing sport, fitness or physical activity in your free time since the lock- down? (in hours)	0 = 1 hour or less, 1 = >1 to 2 hours, 2 = >2 to 3 hours, 3 = >3 to 4 hours, 4 = more than 4 hours, 77 = I don't know
SingleChoice	true	sport3	Yes	How has your overall physical activity changed since the lockdown has been imposed?	1 = Increased, 2 = Stayed the same, 3 = Decreased, 77 = I don't know
SingleChoice	true	platf1	Yes	Do you currently use platforms or tools to do sports at home?	0 = No, 1 = Yes, 77 = I don't know
MultipleChoice	true	platf2	Yes	Which of the following platforms or tools do you currently use to do sports at home?	1 = YouTube, 2 = Special websites with workout videos, 3 = Live-trainings/Live- streams, 4 = Home-Trainer, 5 = Others, 6 = I don't use any platforms or tools
Knob	false	Heigh	No	How tall are you when you are not wearing shoes? (In cm) (Similar in National Health and Nutrition Examination Sur- vey (NHANES) [9])	Integer
Knob	false	weigh1	No	How much do you weigh when you are not wearing any clothes and shoes? (In kg) (Similar in NHANES12)	Integer
Questiontype	Required	Label	In Follow- Up?	Question	Encoding and Answeroptions
SingleChoice	true	weigh2	Yes	How has your weight changed since the lockdown has been imposed mid of March?	1 = Increased, 2 = Decreased, 3 = Un- changed, 77 = I don't know
SingleChoice	true	hyper1	Yes	Have you ever been diagnosed with high blood pressure, a so called hyperten- sion, by a physician? If you do not have high blood pressure, please click “No response” in the following ques- tions. (Similar in National Health In- terview Survey (NHIS) [7])	0 = No, 1 = Yes, 99 = No repsonse
SingleChoice	true	hyper2	Yes	Are you currently taking blood pressure lowering medication? (Similar in NHIS9)	0 = No, 1 = Yes, 99 = No repsonse
SingleChoice	true	hyper3	No	Have you measured your blood pressure before the lockdown has been imposed?	1 = Increased, 2 = Decreased, 3 = Un- changed, 77 = I don't know, 99 = No response
SingleChoice	true	hyper4	Yes	Do you currently measure your blood pressure?	0 = No, 1 = Yes, 99 = No response
SingleChoice	true	hyper5	Yes	How have your blood pressure values changes since the lockdown has been imposed?	1 = Increased, 2 = Decreased, 3 = Un-changed, 77 = I don't know, 99 = No response
SingleChoice	true	diabe1	Yes	Have you ever been diagnosed with diabetes mellitus by a physician? (gesta- tional diabetes is not included) (Similar in BRFSS [6])	0 = No, 1 = Yes, 99 = No response
SingleChoice	true	diabe2	Yes	Are you currently taking blood sugar lowering medication? (Similar in NHIS9)	0 = No, 1 = Yes, 99 = No response
SingleChoice	true	diabe3	No	Have you measured your blood sugar levels before the lockdown has been imposed?	0 = No, 1 = Yes, 99 = No response
SingleChoice	true	diabe4	Yes	Do you currently measure your blood sugar levels?	0 = No, 1 = Yes, 99 = No response
SingleChoice	true	diabe5	Yes	How have your blood sugar levels changes since the lockdown has been imposed?	1 = Increased, 2 = Decreased, 3 = Unchanged, 77 = I don't know, 99 = No response
SingleChoice	true	blood1	Yes	Have you ever been diagnosed with high blood lipids or high cholesterol by a physician? (Similar in NHIS [7])	0 = No, 1 = Yes, 99 = No response
SingleChoice	true	blood2	Yes	Are you currently taking medication for high cholesterol? (Similar in NHIS [7])	0 = No, 1 = Yes, 99 = No response
SingleChoice	true	blood3	Yes	How have your blood lipid levels changes since the lockdown has been imposed?	1 = By myself, 2 = On the advices of relatives or acquaintances, 3 = By the physician, 99 = No response
SingleChoice	true	medic1	Yes	Have one or more of your planned medical visits to a family doctor or resident specialist been postponed or canceled since the lockdown has been imposed mid of March?	0 = No, 1 = Yes, 3 = No appointments were scheduled, 77 = I don't know
SingleChoice	false	medic2	Yes	If yes: By whom has the postponement or cancellation of the doctor's visit been initiated?	1 = By myself, 2 = On the advices of relatives or acquaintances, 3 = By the physician, 99 = No response
Questiontype	Required	Label	In Follow- Up?	Question	Encoding and Answeroptions
SingleChoice	true	physi1	Yes	Has your treatment with a physiotherapist, speech therapist or occupational therapist been postponed or canceled since the lockdown has been imposed mid of March?	0 = No, 1 = Yes, 3 = No need for treatment, 77 = I don't know
SingleChoice	false	physi2	Yes	If yes: By whom has the postponement or cancellation of the doctor's visit been initiated?	1 = By myself, 2 = On the advices of relatives or acquaintances, 3 = By the physician, 99 = No response
SingleChoice	true	hospi1	Yes	Has an examination or treatment in a hospital been postponed or canceled since the lockdown has been imposed?	0 = No, 1 = Yes, 3 = No need for examinations or treatment, 77 = I don't know
SingleChoice	false	hospi2	Yes	If yes: By whom has the postponement or cancellation of the doctor's visit been initiated?	1 = By myself, 2 = On the advices of relatives or acquaintances, 3 = By the physician, 99 = No response
SingleChoice	true	medic3	Yes	Has it been more difficult for you to get the medicines you normally take since the lockdown has been imposed mid of March (e.g. due to longer delivery times or other effects of the restrictions)?	0 = No, 1 = Yes, 3 = I don't take any medication, 77 = I don't know
SingleChoice	true	pain1	Yes	Have you had pain other than or more severe than usual pain in the past 2 weeks? (Similar in Brief Pain Inventory (BPI)13)	0 = No, no pain, 1 = Yes, mild to moderate pain, 2 = Yes, severe to very severe pain, 99 = No response
SingleChoice	true	pain2	Yes	Now it's about the effects of this pain: To what extent have you been affected by this pain in your daily life, leisure time and professional activities in the past 2 weeks?	0 = No impairment, 1 = Mild to moderate impairment, 2 = Severe to very se- vere impairment, 3 = I did not have any pain, 99 = No response

### 4.1 The questionnaires

The PHA study within our Corona Health app consists of two questionnaires: baseline, and follow-up. With the consent of the participants, we additionally gathered mobile sensing data.

### 4.2 Baseline questionnaire

The initial section of the baseline questionnaire gathers basic demographic information from the participants, including their gender, age, and country of residence. The rest of the questionnaire is divided in the following sections encompassing 69 questions in total.

1. **Medical History:** Questions about any comorbidities experienced in the past 12 months.
2. **Cardiovascular risk factors:** Questions addressing various lifestyle factors associated with cardiovascular health, including alcohol and tobacco usage, dietary habits, physical activity levels, and body metrics including height and weight.
3. **Cardiovascular diseases and access to medical care:** Questions about pre-existing comorbidities including diabetes mellitus, hypercholesterolemia, hypertension, and assessment of access to medical care since the start of the pandemic, including frequency of doctors’ appointments and availability of medications.
4. **Physical Pain:** Questions regarding occurrences of temporary physical pain, such as headaches or toothaches.

### 4.3 Follow-up questionnaire

The follow-up questionnaire explores changes in the previously assessed topics since the completion of the last questionnaire, comprising a total of 36 questions.

### 4.4 Mobile sensing data

Corona Health gathers mobile sensing data pertinent to research on the physical and mental health of the population amidst the COVID-19 pandemic. Data are collected in the domains of device information (for both Android and iOS), approximate location data during the completion of questionnaires (for both Android and iOS), and consolidated application usage statistics (exclusively by Android).

### 4.5 Dataset overview

**Fig. 2:**
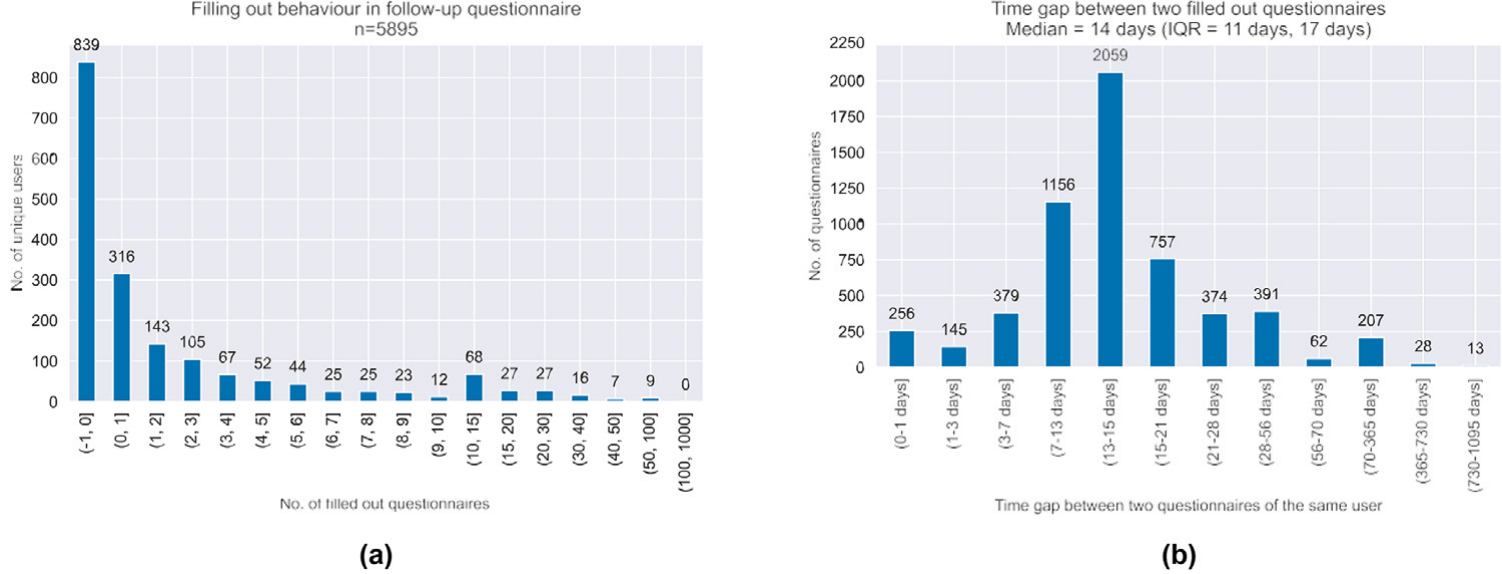
Variability in response patterns within our convenient EMA sampling. (a) User engagement in follow-up questionnaires: Nearly half of all participants did not complete any follow-up questionnaires. User participation exhibits an exponential decline, with a noticeable peak in the interval of 10 to 15 completed questionnaires. (b) Interval between successive questionnaire submissions: The distribution of time gaps between questionnaire submissions by the same user appears to be normally distributed. While some users complete the subsequent questionnaire earlier, others do so later than the intended 14-day interval.

**Fig. 3:**
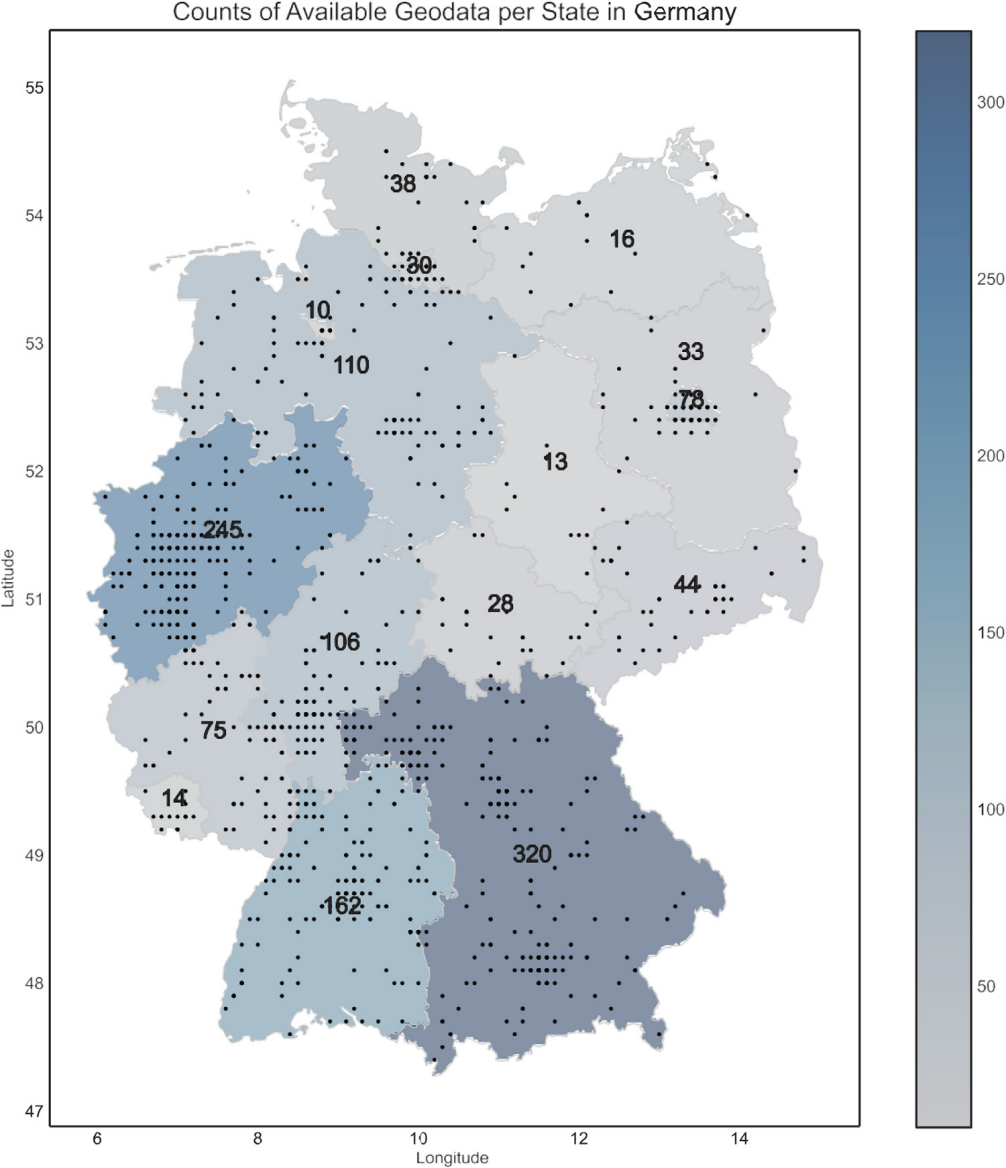
Geospatial data overview for Germany's baseline questionnaire (n=1322), displaying user locations during responses. The heatmap illustrates sample density per state; darker shades represent higher concentrations.

The dataset comprises information on baseline and iteratively filled follow-up questionnaires between 2020-07 and 2023-10. Baseline characteristics of the 1805 participants are provided in Table 1. The available geodata is visualized in Fig. 3. Most participants were male (63.3 %) with a median age of 40 years. Nearly all (98.7 %) were located in Germany when completing the questionnaire. A notable challenge encountered in the analysis of convenient EMA data arises from the variability in the timing of responses. While the study design prescribes a 14-day interval between consecutive questionnaires for each participant, the actual practice diverges significantly. Participants often completed follow-up questionnaires at their discretion, leading to an observed median interval of 14 days, and an interquartile range of 11 and 17 days (details given in Fig. 2). This variability in response timing is an important consideration in the analysis and interpretation of the data. Another critical dimension of our study involves the exponential attrition rate observed among participants. Notably, 46 % of the users did not complete a second questionnaire, and merely 8.5 % (representing a total of 154 users) submitted more than 11 questionnaires. Similar patterns of participant engagement have been documented in other investigations within the Corona Health project [10,11]. The analysis revealed distinct regional variations in user engagement across German states (see Fig. 3). Western states showed a higher proportional user engagement than Eastern states. Bavaria had the highest engagement (0.024 ‰), while Saxony-Anhalt had the lowest (0.006 ‰). When sorted by engagement, the top 10 states were all in the West, while the bottom 6 were in the East. The lessons from the challenges posed by such data structure are further discussed in the next section.

## Limitations

The study faces several limitations that warrant consideration. One significant issue is selection bias, as the *Corona Health* app relies on voluntary participation from smartphone users, likely skewing the sample towards more technologically adept individuals. This limits the generalizability of the findings to the broader population. Additionally, the study experienced high dropout rates, with only 38.4% of the 7,290 verified app-users completing any questionnaires of any study, and just 52.7% of those continuing with follow-up surveys. Such attrition poses challenges for the reliability of longitudinal data. Another limitation is the lack of initial comprehensive demographic data collection in some studies, which restricts subgroup analyses. Data granularity was compromised by coarsening location information to a precision of 11.1 km to ensure privacy, which limits fine-grained geographic analyses. Furthermore, the app usage statistics feature was available only for Android users, leading to inconsistencies in data collection between platforms. The app also primarily relied on self-reported data and a limited set of sensor metrics, such as location and app usage, without incorporating more diverse physiological measures.

The feedback mechanism employed in the app could benefit from a more complex rule engine to allow for dynamic and personalized feedback, potentially enhancing user engagement. The stringent adherence to *Medical Device Regulations* (MDR) and *General Data Protection Regulation* (GDPR) ensured privacy but may have slowed development and limited feature inclusion. Critically, the lack of an engaging onboarding process might have contributed to the high dropout rates; future iterations could address this by incorporating interactive tutorials or gamification elements. Although EMA reduces recall bias, the reliance on self-reported data and high dropout rates challenge the robustness of longitudinal analysis. The coarse granularity of location data and the absence of additional objective health metrics, such as physical activity or sleep, limit the depth of insights that can be derived.

## Code Availability

The code is publicly available to anyone on GitHub: github.com/joa24jm/CH-Heart.

## Ethics Statement

The study was conducted in accordance with the *German Medical Device Regulations* (MDR) and the *General Data Protection Regulation* (GDPR). Ethical approval was obtained from the University of Würzburg's Ethics Committee (approval no. 130/20-me). Data were anonymized, and participants’ privacy was safeguarded throughout the study.

## Credit Author Statement

**Johannes Allgaier:** Conceptualization; Methodology; Software; Data Curation; Formal Analysis; Visualization (figures and tables); Writing - Original Draft; Writing - Review & Editing. **Felizitas Eichner:** Writing - Review & Editing; Validation; Investigation. **Stefan Störk:** Writing - Review & Editing; Validation; Investigation. **Peter Heuschmann:** Supervision. **Rüdiger Pryss:** Supervision; Project Administration; Conceptualization; Writing - Review & Editing; Funding Acquisition.

## Institutional Review Board Statement

Data was collected using the Corona Health app. The Corona Health app study was conducted in accordance with the German medical products law. The data protection officer and the ethics committee of the University of Würzburg, Germany, approved the study (No. 130/20-me). The procedures used in this study adhere to the tenets of the Declaration of Helsinki.

## Data Availability

ZenodoPhysical Health of Adults during Covid-19 (Original data).

## Appendix Supplementary materials

Image, application 1
